# Vitamin D Receptor Is Necessary for Mitochondrial Function and Cell Health

**DOI:** 10.3390/ijms19061672

**Published:** 2018-06-05

**Authors:** Chiara Ricca, Alessia Aillon, Loredana Bergandi, Daniela Alotto, Carlotta Castagnoli, Francesca Silvagno

**Affiliations:** 1 Department of Oncology, University of Torino, Via Santena 5 bis, 10126 Torino, Italy; chiara.ricca48@edu.unito.it (C.R.); alessia.aillon@edu.unito.it (A.A.); loredana.bergandi@gmail.com (L.B.); 2 Department of Chirurgia Generale e Specialistiche, Banca della Cute, AOU Città della Salute e della Scienza Torino, Via Zuretti 29, 10126 Torino, Italy; daniela.alotto@gmail.com (D.A.); ccastagnoli@cittadellasalute.to.it (C.C.)

**Keywords:** vitamin D receptor, silencing, mitochondria, reactive oxygen species, respiratory chain, cytochrome C, cell proliferation, cell death

## Abstract

Vitamin D receptor (VDR) mediates many genomic and non-genomic effects of vitamin D. Recently, the mitochondrial effects of vitamin D have been characterized in many cell types. In this article, we investigated the importance of VDR not only in mitochondrial activity and integrity but also in cell health. The silencing of the receptor in different healthy, non-transformed, and cancer cells initially decreased cell growth and modulated the cell cycle. We demonstrated that, in silenced cells, the increased respiratory activity was associated with elevated reactive oxygen species (ROS) production. In the long run, the absence of the receptor caused impairment of mitochondrial integrity and, finally, cell death. Our data reveal that VDR plays a central role in protecting cells from excessive respiration and production of ROS that leads to cell damage. Because we confirmed our observations in different models of both normal and cancer cells, we conclude that VDR is essential for the health of human tissues.

## 1. Introduction

The active form of vitamin D (1,25(OH)_2_D_3_) exerts its beneficial effects on the whole organism by regulating calcium homeostasis and by modulating a large set of genes involved in the differentiation and function of virtually every tissue. The transcriptional control is mediated by the vitamin D receptor (VDR), which promotes ligand-dependent induction or repression of gene transcription together with its binding partner retinoid X receptor (RXR) and many recruited activators or repressors. The incredibly high number of target genes accounts for the pleiotropic functions of VDR.

Besides the long-recognized role of 1,25(OH)_2_D_3_ in regulating calcium and phosphate metabolism, many biological networks are influenced by VDR, including bone remodeling [1], xenobiotic detoxification [2], cell physiology (reviewed in [3,4]), immunity [5], and metabolism [6,7,8]. Recently, a novel mitochondrial localization of VDR has been described [9,10], and the characterized mitochondrial function of 1,25(OH)_2_D_3_/VDR has been depicted as the hub linking the control of cell metabolism to the transcriptional status of the cell. In fact, the work of our group [11,12] and others [13] has demonstrated that, through VDR activity, 1,25(OH)_2_D_3_ reduces mitochondrial respiration and rewires cell metabolism toward the biosynthetic pathways. This metabolic control sustains both the proliferative asset [11] and more specialized functions of the cells [12], depending on the cellular phenotype. On the basis of these recent observations, it is clear that not only the defective activity of VDR affects the expression of many genes, and thus the levels of many proteins, but also mitochondrial metabolism and function must be profoundly altered by VDR failure. The mitochondrial compartment is central in many processes; besides being the powerhouse of the cell, mitochondria are also important reservoirs of metabolic intermediates, are considered calcium and iron stores [14,15], and behave as molecular factories (for example they are the site of iron insertion in organic molecules). Given their central role, a severe mitochondrial damage leads to apoptotic cell death.

The aim of this work was to explore the results of a defective expression of VDR in cell health and function. We silenced the receptor in different cell types and observed a severe reduction in cell proliferation followed by cell death. We investigated the molecular mechanisms governing the increased vulnerability of the silenced cells and demonstrated the involvement of the mitochondrial compartment.

## 2. Results

### 2.1. Two Different Human Cell Lines and Human Primary Cells Silenced for VDR Strongly Reduce Their Proliferation Rate

**Figure 1:**
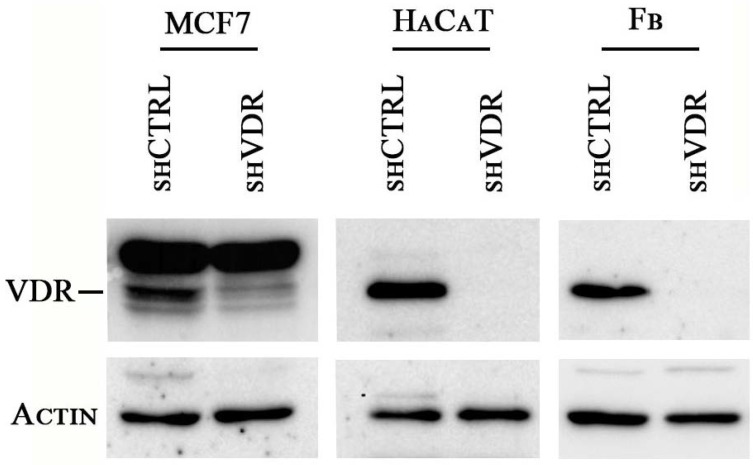
shRNA-mediated Vitamin D receptor (VDR) knockdown in the human cells MCF7, HaCaT, and primary fibroblasts (Fb) abrogates VDR expression. The cells were silenced by lentiviral infection with an shRNA against VDR (shVDR) or with a scrambled non-targeting shRNA as control (shCTRL). Seven days after infection, VDR expression was evaluated in the cellular whole extracts by western blot analysis. Actin was detected as an internal control for protein loading.

**Figure 2:**
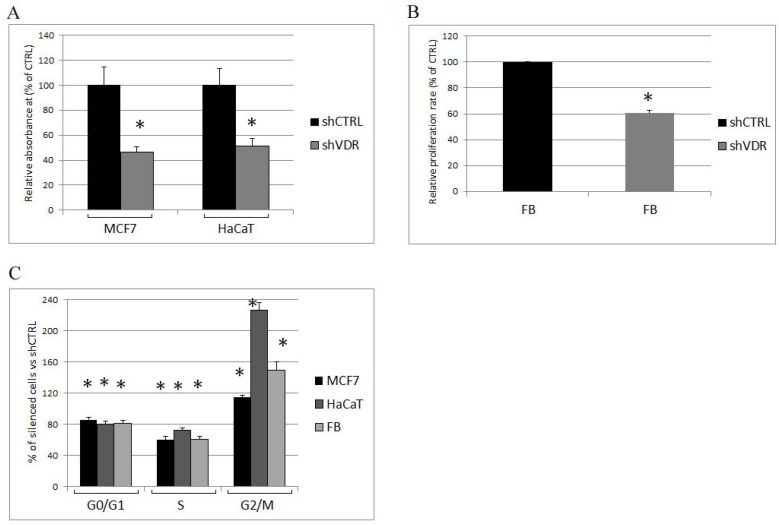
Analysis of cell proliferation in silenced cells. One week after infection, the control (shCTRL) and VDR knockdown cells (shVDR) were seeded and assayed for (**A**) proliferation rate, measured by crystal violet staining or (**B**) BrdU incorporation; (**C**) The cell cycle of MCF7, HaCaT, and fibroblasts (Fb) was evaluated by cytofluorimetry, and the distribution of the silenced cells throughout the cell cycle was expressed as percentage of the shCTRL cells in the same phase. The data are expressed as the means ± SD of three independent experiments; * *p* < 0.05 compared to the control.

With the aim of investigating the effects of a severe reduction of VDR activity on cell physiology, we silenced the receptor by lentiviral delivery of shRNA against human VDR. Three different cell types were selected as examples of malignant, non-malignant, and healthy phenotypes: the human breast cancer cell line MCF7, the human proliferating keratinocyte cell line HaCaT, and primary healthy human fibroblasts, respectively. The genetic ablation of VDR expression by this technique was very effective, as previously demonstrated [11], and the suppression of the protein was confirmed in all cell types by western blotting analysis. The abatement of VDR expression is shown in Figure 1. One week after infection, cell proliferation was investigated either by crystal violet staining (MCF7 and HaCaT cells) or by BrdU incorporation (fibroblasts), and the results are presented in Figure 2A,B. In all cell types, the silencing of VDR caused a great reduction of growth. Accordingly, the analysis of their cell cycle showed a remarkably reduced S phase and a decreased G0/G1 phase, and the cells accumulated in the G2/M phase (Figure 2C).

### 2.2. The Ablation of VDR Enhances Mitochondrial Respiratory Activity and the Production of Reactive Oxygen Species

**Figure 3:**
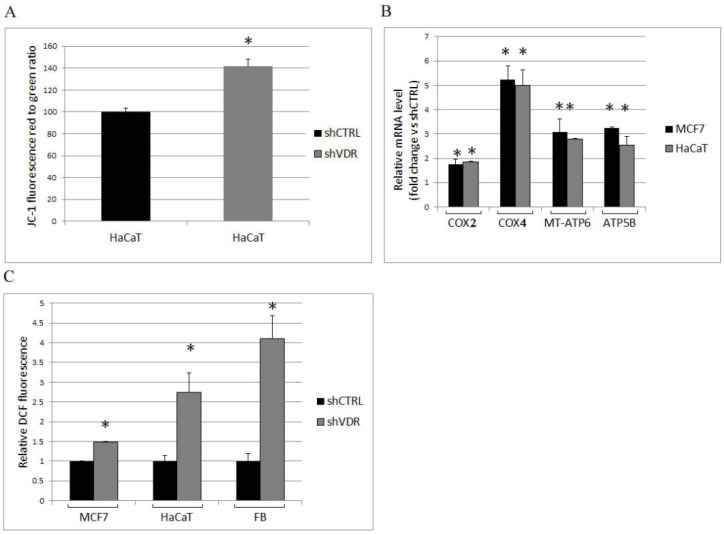
The silencing of VDR induces mitochondrial respiration and enhances the production of ROS. The metabolic assays and the extraction of mRNA were carried out one week after silencing the cells with shRNA control (shCTRL) or VDR shRNA (shVDR). (**A**) The mitochondrial respiratory activity was assessed in HaCaT cells by cytofluorimetric evaluation of the mitochondrial dye JC-1, and (**B**) the expression of the respiratory chain complexes was analyzed by real-time PCR. The values plotted on the graph represent the fold change in transcript expression in silenced versus control cells and are displayed as the means ± SD of three independent experiments; (**C**) reactive oxygen species (ROS) production was measured and expressed relative to control cells. The data represent the means ± SD of three independent experiments; * *p* < 0.05 compared to the control.

We have previously demonstrated that VDR controls mitochondrial respiratory activity [11]. Here, we confirmed that VDR silencing enhanced the respiratory activity of HaCaT cells by measuring the increment of mitochondrial membrane potential (Figure 3A); moreover, by real-time PCR analysis of MCF7 and HaCaT transcripts, we detected the increased expression of several components of the respiratory chain coupled to oxidative phosphorylation. Because both nuclear- and mitochondrial-encoded proteins are required for the formation of active respiratory complexes, we evaluated the transcription of two subunits of cytochrome C oxidase (COX or respiratory complex IV) and two subunits of ATP synthase whose transcripts are both of mitochondrial and nuclear origin: COX2 and MT-ATP6 (a mitochondrial gene encoding the ATP synthase Fo subunit 6) are markers of mitochondrial transcription activity, and COX4 and ATP5B (a nuclear gene encoding ATP synthase subunit beta) are markers of the nuclear contribution to respiratory chain modulation. Their increased expression, shown in Figure 3B, was in agreement with the observed enhanced respiratory membrane potential. One of the consequences of the respiratory burst is the production of reactive oxygen species (ROS); therefore, we measured ROS production in control cells and VDR knockdown cells. We demonstrated the increase of ROS levels in all silenced cells (Figure 3C) and detected the highest increment in primary fibroblasts. 

On the basis of our observations, we concluded that, in all cell types analyzed, VDR was an essential negative modulator of mitochondrial respiration, and its ablation increased both the expression and the activity of the respiratory chain, and the consequent ROS production.

### 2.3. The Silencing of VDR Triggers Long-Term Cellular Damage and Cell Death

**Figure 4:**
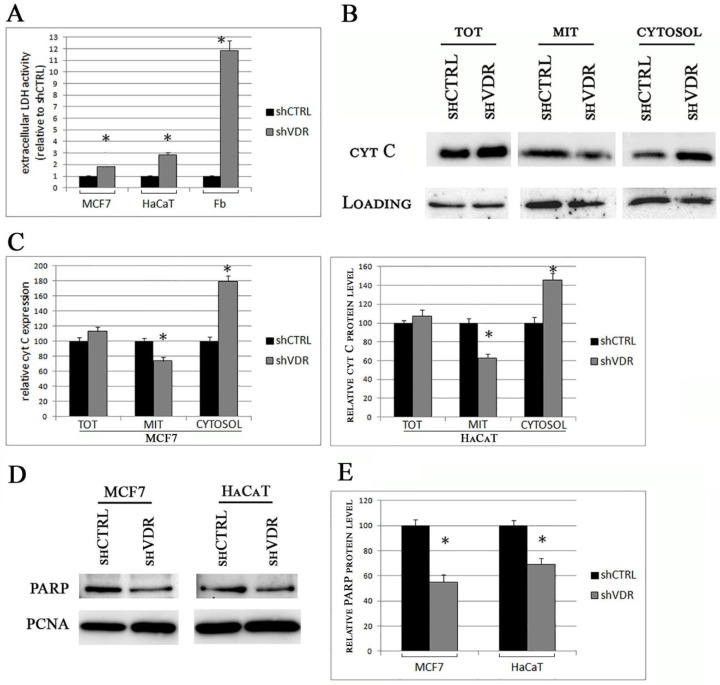
VDR silencing leads to a long-term cellular damage, loss of mitochondrial integrity, and apoptotic death. Two weeks after silencing, cell damage was evaluated. (**A**) Toxicity was assayed by lactate dehydrogenase (LDH) release in the extracellular medium; (**B**) The intracellular levels of cytochrome C were evaluated by western blotting analysis of total extracts (TOT), mitochondrial (MIT), and cytosolic fractions. Actin expression was used as a control of equal loading in total and cytosolic extracts, whereas equal mitochondrial loading was verified by VDAC detection; (**C**) Bands from three different experiments were quantified and normalized for loading, and the data were plotted on graph as percentage of control; (**D**) Full-length PARP (116 kDa) was detected in nuclear extracts of control (shCTRL) or silenced cells (shVDR) by western blotting analysis, and PCNA was used as a loading control. The blots are representative of three independent experiments; (**E**) The bands were quantified and normalized for loading, and the data were plotted on graph as percentage of shCTRL. The data represent the means ± SD of three independent experiments; * *p* < 0.05 compared to the control.

Two weeks after delivery of shRNA particles, the silenced cells lost their healthy phenotype and looked damaged when observed under the microscope. We hypothesized a massive apoptotic death caused by the measured increase of ROS; therefore, we decided to quantify the cellular damage and to verify the mitochondrial origin of the death process. First, we assessed the release of the enzyme lactate dehydrogenase (LDH) as a marker of lost cell integrity. The results of our analysis are displayed in Figure 4A. As expected, all the silenced cells accumulated great amounts of LDH in their supernatants in comparison to control cells. This increase in LDH was particularly evident in the medium of the silenced fibroblasts. Next, we evaluated the signs of the lost mitochondrial integrity by western blotting analysis of cytochrome C content in subcellular fractions. Total lysates were prepared along with mitochondrial and cytosolic extracts, and the levels of cytochrome C were quantified with a specific antibody. The results of our analysis are displayed in Figure 4B, and the data were quantified and plotted in Figure 4C. Both in MCF7 and in HaCaT cells, we found a decreased content of cytochrome C in the mitochondrial compartment and, alongside this loss, we detected the increase of the mitochondrial protein in the cytosolic fractions. At the same time, the expression of cytochrome C in the whole lysates was unchanged, demonstrating the release of the mitochondrial protein pool into the cytosolic milieu. The intracellular trafficking of an essential element of the respiratory chain is considered the hallmark of apoptosis driven by a defective mitochondrial function. We also investigated another marker of an ongoing apoptotic death: the cleavage of the nuclear enzyme poly ADP ribose polymerase (PARP). In fact, the proteolytic cleavage of PARP into 89 and 24 kDa fragments by caspases is an early indicator of apoptosis [16]. We analyzed the protein content of nuclear preparations from control and silenced MCF7 and HaCaT cells and were able to detect a decreased amount of the 116 kDa PARP protein in the nuclear extracts of silenced cells, which was the demonstration of the occurring cleavage and loss of the full-length enzyme (Figure 4D). The results of these experiments were quantified and plotted on graph, as shown in Figure 4E.

All together, our data demonstrated that the silencing of VDR led to a severe cell damage that had all the signs of a mitochondrial-mediated apoptotic death.

## 3. Discussion

Vitamin D is active in every tissue, and the perturbation of its signaling is involved in many diseases [17]. The importance and the pleiotropic effects of the activity of its receptor VDR have been elucidated by the murine knockout models, which have displayed a modified phenotype in many tissues, such as bone, intestine (reviewed in [18]), skin [19], lung [20], muscle [21], endothelium [22], and adipose tissue, and in metabolism [23,24]. Although the knockout models are useful to investigate the general pathways controlled by vitamin D, at the cellular level they have a major flaw: they originate cells that, since their formation and embryonic development, have never relied on VDR for transcriptional control and modulation of metabolism. If we wonder about the importance of VDR activity and whether it is essential for cellular tasks, another approach is to knockdown the receptor in a cell population that expresses VDR to modulate growth, differentiation, and many other functions. When the deranged signaling of 1,25(OH)_2_D_3_ is investigated, it must be considered that the insufficiency of vitamin D and the loss of the receptor have some similar but partly distinct consequences. The hypocalcemic phenotype shared by vitamin D deficiency and VDR knockout models can be reversed by a high-calcium diet [25], but even at very low levels of 1,25(OH)_2_D_3_, the VDR can still operate in a ligand-independent modality [26,27] or can respond to other molecules [28,29,30]. Therefore, in order to analyze fully and unambiguously the perturbation of 1,25(OH)_2_D_3_ signaling, the best approach is to delete the receptor, and we followed this line of investigation. 

In this work, we carried out a genetic silencing of VDR in three cell types different from each other, for the purpose of testing the general importance of VDR in cell physiology. MCF7 were selected as an example of malignant human cancer cell lines, HaCaT cells were a good model of proliferating but not transformed human keratinocytes, and primary human fibroblasts were chosen as a model of healthy human cells.

The first important finding of this work was that the ablation of the receptor resulted in increased respiratory activity that enhanced the production of intracellular ROS. Interestingly, in all cell types, we found that VDR controls both the mitochondrial (COX2 and MT-ATP6) and the nuclear transcription (COX4 and ATP5B) of the proteins involved in respiratory activity and ATP synthesis, in agreement with the necessity of coordinating the nuclear and the mitochondrial transcription of the components of the respiratory process. It is known that the respiratory chain is a major source of ROS; in particular, the complexes I, III, and IV are involved in radical biosynthesis [31,32]. ROS production is beneficial to some extent and is involved in cell cycle progression [33], but an excessive boost can be detrimental and can trigger cell damage. In all three silenced cellular models, the increase in ROS levels was remarkable, especially in primary fibroblasts, and could exceed the antioxidant defenses. Indeed, while the initial effect of silencing was growth arrest and the modulation of the cell cycle, the long-term effect of VDR loss was cell damage, measured as LDH release. The increase of ROS levels and the toxicity were directly proportional, since we observed the smallest rise of ROS and toxicity in silenced MCF7 and the highest effects in silenced primary fibroblasts. This observation is reasonably accounted for by some reported characteristics of cancer cells. In fact, it is known that the transformed cells use ROS signals to drive proliferation and other events required for tumor progression and that the elevated ROS levels are balanced by an increased activity of antioxidant enzymes in cancer cells [34]; accordingly, the protective role of VDR was particularly evident in healthy fibroblasts, although, in our experimental setting, VDR defended even the most transformed MCF7 cells against oxidative stress.

In our previous work, we demonstrated the metabolic importance of VDR and its effect on proliferation: the receptor curbs the respiratory activity and allows the rewiring of metabolic intermediates toward biosynthesis, thus sustaining proliferation [11]. The results of the present research unveil a novel role for VDR in cellular physiology, namely, the protection from the excessive respiratory activity and the limitation of ROS production. In this study, we observed the negative effects of the derangement of the metabolic control exerted by VDR; indeed, in the long run, the excessive production of ROS consequent to VDR ablation had deleterious effects on the mitochondrial function and survival of cells. One of the consequences of the excessive damage caused by ROS is the variation in mitochondrial membrane permeability that results in cytochrome C release and apoptotic death [35]. In line with this, we demonstrated that, after two weeks of absence of VDR, mitochondrial integrity was lost, and the cells showed the signs of an apoptotic fate. The interplay between vitamin D–VDR, ROS signaling, and the antioxidant system is complex; on the one hand, it has been demonstrated that vitamin D and its analogues can increase the cytotoxicity mediated by ROS [36], while, on the other hand, few reports have proved that vitamin D–VDR is able to inhibit the apoptosis triggered by oxidative stress [37,38,39,40,41]; in addition, in this study, for the first time, we showed the protective role exerted by VDR itself, without additional stressors. Moreover, our study investigated a novel mechanism involved in the antiapoptotic effects of VDR, previously ascribed only to its transcriptional activity [42]. In fact, not only the loss of VDR disrupts the traditional pathways regulated by its transcriptional control [42], but also, as we demonstrated, the silencing of VDR generates an unbalanced metabolism that leads to cytotoxicity. Although we have displayed the essential role of VDR in cell metabolism, health, and survival, our data and conclusions are not necessarily in contrast with the fact that the VDR knockout phenotype in animals is not lethal. Obviously, the tissues lacking VDR since their embryonic development have found compensatory mechanisms to balance the effects of VDR deletion.

In conclusion, in the present study, we discovered a novel important role for VDR in cell health. We demonstrated that the mitochondrial effects of the receptor not only regulate the respiratory activity but also protect from oxidative damage and preserve mitochondrial integrity and cell survival. Our data were obtained in different cell types, cancerous as well as healthy cells, rendering the discovered novel function a general feature of vitamin D–VDR role in many tissues. 

Another intriguing consideration about this study is based on the fact that the phenotype obtained by the experimental silencing of the receptor could mimic the pathological situations in which the expression of VDR is downregulated (for example by epigenetic mechanisms [43,44]) or its activity is compromised (for example by polymorphisms [45,46]). It is interesting to highlight that many respiratory chain dysfunctions and deleterious ROS overproduction are recurrent themes in human pathologies, ranging from neurodegenerative diseases to cancer [47,48], and may be of paramount importance in ageing [49]. The results of this study demonstrate the protective role of VDR and raise the possibility that the loss of VDR function could be partly responsible of, or at least could be an adverse event in such diseases.

## 4. Materials and Methods

### 4.1. Cell Culture

The immortalized human epidermal keratinocyte cell line (HaCaT) and the MCF7 human breast cancer cell line were purchased from American Type Culture Collection (ATCC) (Manassas, VA, USA). Dermal primary fibroblasts from healthy donors were obtained from Banca della Cute, Turin, Italy, and were used in early passages. The cells were cultured in Dulbecco’s modified Eagle’s medium (DMEM) that had been supplemented with 10% fetal bovine serum and 1% antibiotics (penicillin, streptomycin), at 37 °C in a humidified atmosphere containing 5% CO_2_. All culture reagents were from Sigma-Aldrich (Sigma, St. Louis, MO, USA).

### 4.2. Lentiviral-Mediated shRNA Targeting

PLKO.1 lentiviral shRNA clones targeting the human VDR (TRCN0000276543) and a scrambled nontargeting control were purchased from Sigma (Sigma Mission shRNA) (Sigma, St. Louis, MO, USA) and were previously described and characterized in terms of efficiency [11]. Lentiviral transduction particles were produced in HEK293T cells as previously reported [11]. Briefly, the cotransfection of the shRNA plasmid together with the packaging vectors was carried out by lipofectamine reagent, and the supernatants were used for overnight transduction of the cells. Puromycin selection began 24 h after infection. Within one week from infection or after two weeks, the cells were seeded for experimental assays or harvested for RNA and protein analysis.

### 4.3. Extract Preparation and Western Blotting Analysis

Subcellular fractionation and Western blotting analyses were conducted as previously described [10]. The protein content of the total extracts and mitochondrial fractions was quantified using the DC protein assay (Bio-Rad, Hercules, CA, USA); 50 µg of total lysates and 30 µg of the mitochondrial or nuclear fractions were separated by 10% SDS-PAGE and analyzed using Western blotting. The analysis of cytochrome C was carried out after a 12% SDS-PAGE. The proteins were immunostained with the indicated primary antibodies for 1 h at room temperature, and detection of the proteins of interest was performed using peroxidase-conjugated secondary antibodies (Pierce, Rockford, IL, USA), followed by ECL detection (ECL detection kit, Perkin Elmer Life Science, Foster City, CA USA). Mouse anti-VDR (sc-13133), anti-actin (sc-8432), anti-PCNA (sc-56), and rabbit anti-PARP (sc-7150) antibodies were purchased from Santa Cruz Biotechnology (Santa Cruz, CA, USA). The anti-VDAC (anti-porin 31HL) monoclonal antibody was purchased from Calbiochem (La Jolla, CA, USA). The mouse anti-cytochrome C (65981A) antibody was from BD Biosciences Pharmingen (San Diego, CA, USA).

### 4.4. Real-Time Polymerase Chain Reaction (qRT-PCR)

Total RNA was extracted with TRIzol^®^ (Invitrogen, Thermo Fisher Scientific, Waltham, MA, USA). One μg of total RNA was reversely transcribed into cDNA, in a final volume of 20 μL, using the High-Capacity cDNA Reverse Transcription Kit (Thermo Fisher Scientific, Waltham, MA, USA), according to the manufacturer’s instructions. Quantitative PCR was carried out in a final volume of 20 μL, using the SensiFASTTM SYBR^®^ Hi-ROX Kit (Bioline Srl, Trento, Italy) with the following primers:

COX2, fwd 5′-CGACTACGGCGGACTAATCT-3′, rev 5′-TCGATTGTCAACGTCAAGGA-3′; COX4, fwd 5′-CGAGCAATTTCCACCTCTGT-3′, rev 5′-GGTCAGCCGATCCATATAA-3′;

ATP5B, fwd 5′-GTGGGCTATCAGCCTACCCT-3′, rev 5′-CAAGTCATCAGCAGGCACAT-3′; MT-ATP6, fwd 5′-CCAATAGCCCTGGCCGTAC-3′, rev 5′-CGCTTCCAATTAGGTGCATGA-3′; β2M, fwd 5′-AGCAAGGACTGGTCTTTCTATCTC-3′, rev 5′-ATGTCTCGATCCCACTTAACTA-3′. Beta 2-microglobulin β2M was used as an internal control. PCR amplification was one cycle of denaturation at 95 °C for 2 min, 40 cycles of amplification, including denaturation at 95 °C for 5 s and annealing/extension at 60 °C for 30 s. The 2^−ΔΔ*C*t^ method was used to analyze the data.

### 4.5. Proliferation Assay

Within one week from silencing, the effect of VDR silencing on the growth of the different human cells was determined either by colorimetric measurement of cell numbers by crystal violet staining (MCF7 and HaCaT cells) or by BrdU incorporation (primary fibroblasts). The primary human fibroblasts were poorly stained by the crystal violet method, and the detection by spectrophotometer was inadequate to quantify these cells; therefore, the more sensitive BrdU assay was chosen. The same number of control or silenced cells (2000, 1000, or 500 cells per well) was seeded on 96-multiwell plates, and the cells were either cultured for five days and then stained with crystal violet or assayed after two days for BrdU incorporation. At the end of this period, MCF7 and HaCaT cells were fixed for 15 min with 11% glutaraldehyde, and the plates were washed three times, air dried, and stained for 20 min with a 0.1% crystal violet solution. The plates were then extensively washed and air-dried prior to solubilization of the bound dye with a 10% acetic acid solution. The absorbance was determined at 595 nm. The proliferation of primary fibroblasts was evaluated by the Cell Proliferation ELISA BrdU kit (Roche Applied Science, Penzberg, Germany), used according to the supplied instructions. The data collected from twelve wells were averaged for each experimental condition, and each experiment was repeated three times.

### 4.6. Cell Cycle Analysis

Within one week from silencing, the control and silenced cells were seeded at the same density and, after 48 h, were detached in 1 mL PBS-EDTA 5 mM by scraping, collected, and fixed in 70% cold ethanol. After 3 h at −30 °C, the cells were centrifuged at 2000 rpm for 5 min, washed twice with PBS, resuspended in 200 µL MUSE^®^ cell cycle reagent, and then incubated for 30 min in the dark at room temperature. Cellular DNA content was analyzed by Muse Cell Analyzer (Merck S.p.a., Milan, Italy). To quantify the relative percentage of cells in the G0/G1, S, and G2/M phases of the cell cycle, the Muse™ Cell Analyzer software was used. 

### 4.7. Cytofluorimetric Evaluation of the Mitochondrial Membrane Potential

JC-1, a mitochondrial dye that stains the mitochondria in living cells in a membrane potential-dependent fashion, was used as previously reported [11]. Within one week from silencing, HaCaT cells were harvested by trypsinization, washed with PBS, and incubated with JC-1 (2 mg/mL final concentration) at 37 °C for 30 min. After washing, JC-1 accumulation was determined using flow cytometric analysis. The amount of JC-1 retained by 10,000 cells per sample was measured at 530 nm (FL-1 green fluorescence) and 590 nm (FL-2 red fluorescence) using a flow cytometer and analyzed using Cell Quest Alias software. The ratio FL2/FL1 was evaluated to determine the mitochondrial membrane potential.

### 4.8. Measurement of Intracellular ROS Production

After one week from silencing, the cells were harvested and loaded for 15 min with 10 μM 2′,7′-dichlorodihydrofluorescein diacetate (DCFH-DA, Sigma). DCFH-DA is a cell-permeable probe that is cleaved intracellularly by nonspecific esterases to form DCFH, which is further oxidized by ROS to form the fluorescent compound dichlorofluorescein (DCF) [50]. DCF fluorescence was determined at an excitation wavelength of 504 nm and an emission wavelength of 529 nm, using a Packard EL340 microplate reader (Bio-Tek Instruments, Winooski, VT, USA). The fluorescence values were normalized to the protein content and expressed as values relative to the control.

### 4.9. Toxicity Assay (LDH Release)

Two weeks after silencing, cell damage was evaluated by measuring the release of lactate dehydrogenase in the growth medium. The medium was collected, and the cells were harvested by scraping and sonicated on ice with two 10 s bursts. Protein content was quantified by the DC protein assay. Aliquots of growth medium were supplemented with a reaction mixture for the measurement of LDH, as previously described [51]. The enzymatic activity in the extracellular medium was measured spectrophotometrically as absorbance variation at 340 nm (37 °C) and was expressed as µmol NADH oxidized/min/mg cell protein, to normalize the extracellular activity to the cell number. The data were plotted relative to control values. 

### 4.10. Bands Quantification and Statistical Analysis

The bBands from protein electrophoresis were quantified by scanning digital densitometry using an ImageJ software analysis (ImageJ version 1.29, Sun Microsystems Inc., Palo Alto, CA, USA). All data were expressed as mean ± S.D of three independent experiments. Statistical analysis of the data was performed using an unpaired, two-tailed Student’s *t*-test; *p* < 0.05 was considered to be significant.
